# Pierre H.A. Jolivet, the *spiritus rector* of leaf beetle research, with a list of his publications

**DOI:** 10.3897/zookeys.547.6758

**Published:** 2015-12-17

**Authors:** Michael Schmitt, Jorge Santiago-Blay

**Affiliations:** 1 Ernst-Moritz-Arndt-Universität, Allgemeine & Systematische Zoologie, Anklamer Str. 20, D-17489 Greifswald, Germany; 2 Unaffiliated

Since more than half a century, Pierre Hippolyte Auguste Jolivet has been the inspiring head of the community of leaf beetle researchers (Fig. 1). He promoted research on Chrysomelidae not only by his nearly 500 publications (see list below), but even more so by his personal input to the international and European symposia on leaf beetles, and especially by the six volumes he co-edited (nos. 274, 316, 338, 339, 340, 445 of the list below) and the three he (co-)authored (nos. 329, 347, 389).

**Figure 1.**
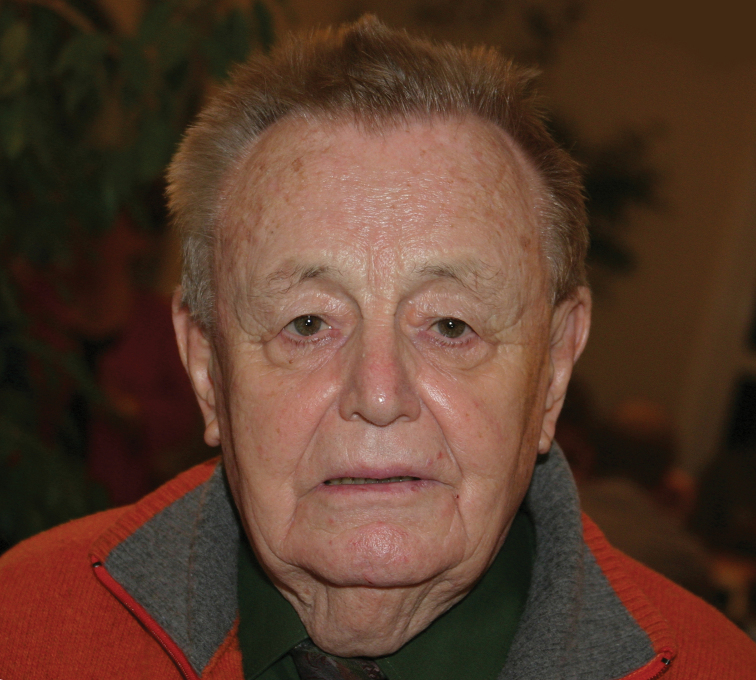
Pierre Jolivet, attending the 50^th^ coleopterists’ meeting in Beutelsbach (near Stuttgart, Germany) on October 27, 2007.

Besides his numerous papers and books on leaf beetles, he has also published on ants, especially ant-plant interactions, on parasites of insects, and on broader topics, such as evolution. In 2005, Pierre initiated the project of a series of volumes, edited by him and the two of us, for which we agreed on the title *Research on Chrysomelidae* (*RoC*). The first two volumes appeared with Brill Academic Publishers, the following volumes as special issues of *ZooKeys* with Pensoft. Now, Pierre has decided to resign from his post as senior editor, and the two of us decided to dedicate the present volume 5 of *RoC* to honour him.

Pierre Jolivet was born on October 12, 1922, two o’clock in the morning, as he reports in his autobiography (Jolivet 2006, no. 421), in Avranches (Manche department, Lower Normandy). He entered the University of Rennes in 1941, where he studied zoology and botany. He received his bachelor in 1943 with a thesis (Diplôme d’Etudes Supérieures) on the biology of the species of *Timarcha*. These beetles fascinated him through the whole of his scientific life, from his student times through his excursions to Brno (Czech Republic) in the 1950s until one of his most recent papers (no. 485). Finally, his autobiography (no. 421) has the title “Mémoires Entomologiques – Paramémoires d’un timarchophile”.

He received the degree of a “Docteur ès Sciences Naturelles” on April 04, 1954 from the Sorbonne, at that time the only university in Paris. There, he produced a thesis in two volumes (nos. 153, 158) on the hind wing morphology, especially venation of Chrysomelidae. In addition, he has studied and depicted the male copulatory organs of representatives of all subfamilies (except Bruchinae). This publication provides an invaluable treasure of basic morphological knowledge, the more because Pierre Jolivet dissected not only specimens of very common species but also of taxa difficult to obtain for students in Europe, e.g. Australian Sagrinae or American Aulacoscelidinae. He had completed the empirical work at the Musée Royal des Sciences Naturelles of Brussels (Belgium), but was supervised by Pierre Grassé at the Sorbonne. In those days in France, one had to produce two theses to obtain the doctoral degree. Pierre Jolivet handed in his second thesis on the leaf beetles of the Balearic Islands (no. 119), also a fundamental contribution to science, in this case to faunistics. No surprise, a considerable section of this second thesis treats extensively the endemic *Timarcha* species, *Timarcha balearica*.

In 1954, Pierre Jolivet undertook the first of his numerous journeys to tropical and exotic areas outside Europe. He travelled through the then Belgian Congo with some detours to Kenya and Uganda, where he studied and collected, of course, leaf beetles. Back in Brussels, he began a series of joint projects with Jean Théodoridès – with whom he had collaborated on leaf beetles before - on gregarines, documented in 15 original papers. These parasites had been recognised as a separate taxon by Pierre Grassé in 1953 in his famous *Traité de Zoologie*.

With a four months expedition to Iran on behalf of the World Health Organisation began Pierre Jolivet’s “United Nations epoch”. His main duty was the scientific supervision of malaria control activities. He worked as a member of several Malaria Advisor Teams for the UN in many countries, e.g. Taiwan, The Philippines, Ethiopia, Sudan, Algeria, Vietnam, and the Cap Verdes, with teaching activities at universities in New Guinea and in Morocco, until his retirement in 1985. In between, he found his wife Madeleine (Mayon), and they married on March 24, 1962. They have two daughters and one son. She accompanied him on many of his trips, and since 1984 it was a familiar sight to see the couple on the occasion of our regular symposia on Chrysomelidae: 1984 in Hamburg (Germany), 1988 in Vancouver (Canada), 1992 in Beijing (China), 1996 in Florence (Italy), 2000 in Iguassu (Brazil), 2004 in Bonn (Germany), 2008 in Durban (South Africa), and 2010 in Budapest (Hungary). From time to time, the two of them also joined the meetings of the German coleopterists at Beutelsbach (near Stuttgart, Germany). Only the last two of our regular symposia had to take place without Pierre and Madeleine Jolivet – the 8th International Symposium on Chrysomelidae in Daegu (South Korea) in 2012, and the 2^nd^ European Symposium on the Chrysomelidae in York (UK).

Out of Pierre’s countless contributions to the understanding of life and evolution of leaf beetles, there is one to be emphasised especially. Together with Joao Vasconcellos-Neto he discovered, described, and named in 1988 a ring defence behaviour of larvae, which they called “cycloalexy” (nos. 276, 319, 477). This newly coined term has made its way into dictionaries of entomology and will always be connected with the name of its discoverers.

Pierre suffered increasingly from health problems in his legs and, since about 2012, did not dare to travel abroad any longer. This year, he decided to step down from the Board of Editors of *Research on Chrysomelidae*. He had initiated this series in 2007, after the three of us had successfully co-operated as editors of the 800 pages volume *New Developments in the Biology of Chrysomelidae*. We shall do our best to make Pierre’s project live on.

We, editors, many authors, and publishers of *Research on Chrysomelidae* are grateful for Pierre’s permanent intellectual stimulation, his helpful input, and his friendship. We wish Pierre and Madeleine all the best, especially a healthy body and mind, and hope that the present issue of *Research on Chrysomelidae* as well as the ones to follow will help to keep up the bond between us, the community of leaf beetle enthusiasts, and Pierre Jolivet, our *spiritus rector*.

Michael Schmitt (michael.schmitt@uni-greifswald.de)

Jorge Santiago-Blay (blayj@si.edu, blayjorge@gmail.com)

## Publications by Pierre Jolivet

**1942**

1. Hybridation probable de deux Chrysomèles: *Chrysomela polita* X *Chrysomela menthastri*. Bulletin de la Société Entomologique de France 47(9): 141.

**1943**

2. Note sur deux Orthoptères rares pour le département de la Manche. Bulletin de la Société linnéenne de Normandie 9(3): 34–35.

3. Sur un coléoptère nouveau pour le département de la Manche: *Chrysomela hyperici* Forst. Bulletin de la Société linnéenne de Normandie 9(3): 60–62.

4. Sur un curieux tropisme de *Steropus madidus* Fabricius. Bulletin de la Société linnéenne de Normandie 9(3): 83–87.

5. *Chrysomela menthastri* et ses variations dans le département de la Manche. Notices, mémoires et documents / Société d‘Agriculture, d‘Archéologie et d‘Histoire Naturelle du Département de la Manche 44: 69–77.

6. Capture de *Stenelmis canaliculatus* et *Anthaxia manca*. Bulletin de la Société Entomologique de France 45(7): 99.

7. Sur un cas de «phorésie» observé chez deux espèces du genre *Timarcha*. Bulletin de la Société linnéenne de Normandie 9 (3): 107–108.

8. *Platyarthrus hoffmanseggi* Br., isopode myrmécophile dans le département de la Manche. Bulletin de la Société linnéenne de Normandie 9(3): 136–137.

9. Capture d’un Triton rare pour la faune normande: Triton (Molge) marmorata Latr. Bulletin de la Société linnéenne de Normandie 9(3): 138–140.

10. Tableau dichotomique pour la détermination des espèces et variétés françaises du genre *Timarcha* Latreille. Miscellanea Entomologica 41(7): 67–72.

11. Jolivet P, Charles Roux: Sur la présence en Bretagne de Microcystis (Clathrocystis) aeruginosa Kutz. Bulletin de la Société Scientifique de la Bretagne 47(3–4): 166–168.

12. Frémy P, Jolivet P: Capture en très grande abondance près de Saint-Lô de *Scapholeberis mucronata*. (O.F. Müller) (Cladocères Daphniides). Bulletin de la Société linnéenne de Normandie 9(3): 47.

**1944**

13. Notes sur quelques espèces et variétés du genre *Timarcha* Latr. Miscellanea Entomologica 41(7): 96–101.

14. De l’hydrotropisme positif de *Steropus madidus*. Miscellanea Entomologica 41(7):102–106.

**1945**

15. Notice biographique sur l’abbé Frémy (1880–1944). Bulletin de la Société Botanique de France 92(4–6): 72–73.

16. Esquisses biogéographiques sur le genre *Timarcha* Latr. Bulletin de la Société linnéenne de Normandie 9 (4): 32–41.

17. Sur la présence *d’Araschnia levana (Lep. Nymphalidae)* dans l’ouest de la France. Bulletin de la Société linnéenne de Normandie 9(4):45–46.

18. Contribution à l’étude des parasites du genre *Timarcha* Latr. Miscellanea Entomologica 42(1): 1–4.

19. A propos du DDT. Miscellanea Entomologica 42(1):13–14.

20. Deux cas de phorésie chez les *Timarcha* (Col. Chrys.). L’Entomologiste 1(8–9): 141.

**1946**

21. Nouveaux cas de phorésie chez les Chrysomélides. L’Entomologiste 2(1): 35.

22. Captures de Puces et de Diptères. L’Entomologiste 2(4):160.

23. La Vie et l’Oeuvre de l’Abbé Frémy (1880–1944). Notices, mémoires et documents / Société d‘Agriculture, d‘Archéologie et d‘Histoire Naturelle du Département de la Manche 55: 1–18.

24. Nouvelle station française et nouvel hôte du Ver *Gordius aquaticus*. Miscellanea Entomologica 42(2): 139–140.

25. Quelques remarques sur l’autohémorrhée chez les *Timarcha* (Col. Chrys.). Miscellanea Entomologica 43(4):29–30.

26. A propos des variations du Doryphore (*Leptinotarsa decemlineata* Say). Miscellanea Entomologica 43(4): 31–33.

27. Sur une larve inédite vivant en parasite dans le sang de *Timarcha tenebricosa* F. Miscellanea Entomologica 43(4): 33–35.

28. Une méthode simple et pratique pour préparer les Grégarines et autres parasites des Insectes. Miscellanea Entomologica 43 (4): 35–36.

29. Sur plusieurs cas de brachélytrie dysplasique dans une population de *Melasoma tremulae* F. Miscellanea Entomologica 43(4): 36–38.

30. Le réflexe d’enfouissement chez *Leptinotarsa* and *Timarcha* (Col. Chrys.). Miscellanea Entomologica 43(4): 38–39.

31. Les parasites des Chrysomélides français (1° liste). Miscellanea Entomologica 43(7):87–92.

32. A propos d’une curieuse anomalie élytrale chez une femelle de *Timarcha tenebricosa* F. Miscellanea Entomologica 43(10): 133–134.

**1947**

33. Notes additives et correctives sur la répartition géographique du genre *Timarcha* Latr. Bulletin de la Société linnéenne de Normandie 9(5):33–34.

34. Le criquet migrateur en Normandie. Bulletin de la Société linnéenne de Normandie 9(5): 134.

35. Une méthode pratique pour la conservation des chenilles: le montage à la gélatine. Revue Française de Lépidoptérologie 11(4): 91–94.

36. Les parasites des Chrysomélides français (2me liste). Miscellanea Entomologica 44(8):108–109.

37. Capture de *Locusta migratoria*, phase grégaire, dans la Manche. L’Entomologiste 3(3): 144.

**1948**

38. Introduction à la Biologie des *Timarcha*. Miscellanea Entomologica 45(1): 1–32.

39. Les Orsodacnidae de la faune française. Miscellanea Entomologica 45(3–4): 33–46.

40. Les parasites des Chrysomélides français. (3me liste). Miscellanea Entomologica 45(5): 52–54.

41. Introduction à l’étude des Gordiacés, Vers parasites d’Insectes. Miscellanea Entomologica 45(9): 83–90.

42. Le rôle des Chrysomélides en pathologie végétale. Miscellanea Entomologica 45(10): 97–99.

43. La conservation de la couleur chez les Cassididae (Col. Chrys.). L’Entomologiste 4(3–4): 140–143.

44. Addenda à la liste des travaux de l’Abbé P. Frémy. Notices, mémoires et documents / Société d‘Agriculture, d‘Archéologie et d‘Histoire Naturelle du Département de la Manche 57: 18.

45. Une «fleur d’eau» à Beuvrigny. Notices, mémoires et documents / Société d‘Agriculture, d‘Archéologie et d‘Histoire Naturelle du Département de la Manche 57: 17.

46. Quelques données récentes sur la biologie des Timarcha Latr. (Col. Chrys.). Miscellanea Entomologica 45(10): 100–102.

47. Contribution à l’étude des *Americanotimarcha* n. subg. (Col. Chrys.). Bulletin du Musée Royal d’Histoire Naturelle de Belgique 24(43): 1–2.

48. La Vie et l’Oeuvre de Constant Houlbert (1857–1947). Miscellanea Entomologica 45(10): 103–108.

49. Les parasites des Chrysomélides français (4me liste). Miscellanea Entomologica 45(10): 109–111.

50. Victor Laboissière (1875–1942). Miscellanea Entomologica 45(11): 103–108.

51. Un nouvel insecticide: le S.N.P. Bulletin de la Société Scientifique de Bretagne 23: 99–110.

**1949**

52. A propos de la coloration chez les Cassididae. L’Entomologiste 5(1–2): 58.

53. Les hybrides d’espèces chez les *Chrysolina* Motsch. L’Entomologiste 5(3–4): 77–80).

54. Les Chrysomélides qui changent de couleur. L’Entomologiste 5(5–6): 190–191.

55. Contribution à l’étude des *Iscadida* Chevrolat (Col. Chrys.). Bulletin de l’Institut Royal des Sciences Naturelles de Belgique 25(40): 1–19.

**1950**

56. A propos des accouplements anormaux des Chrysomélides. L’Entomologiste 6(1): 19.

57. *Bechyneia spinosa*, nov. gen., nov. sp. (Col. Chrys.). Bulletin et Annales de la Société Royale Entomologique de Belgique 86(1–2): 36–39.

58. Sur un cas de trématélytrie chez *Crioceris asparagi* L. (Col. Crioceridae). L’Entomologiste 6(3): 77–79.

59. Un Braconide nouveau pour la faune belge. Bulletin et Annales de la Société Royale Entomologique de Belgique 86(9–10): 203–204.

60. Un accouplement interspécifique chez deux *Chrysolina*. Bulletin et Annales de la Société Royale Entomologique de Belgique 86(9–10): 200.

61. Fluctuations dans une population de Blattes et de Gryllons. L’Entomologiste 6(4–5): 139.

62. Les parasites, prédateurs et phorétiques des Chrysomeloidea (Col.) de la faune franco- belge. Bulletin de l’Institut Royal des Sciences Naturelles de Belgique 26(34): 1–39.

63. *Elytrosphaera melas* n. sp. Bulletin de l’Institut Royal des Sciences Naturelles de Belgique 26(43): 1–4.

64. Jolivet P, Théodoridès, J.: Les Helminthes parasites des Coléoptères Chrysomélides. Annales de la Parasitologie Humaine et Comparée 25(4): 340–349).

65. Une nouvelle espèce de *Timarcha* (Col. Chrys.) des Pyrénées Orientales. Vie et Milieu 1: 235–239.

66. Jolivet P, Théodoridès, J. Découverte d’une espèce nouvelle de *Timarcha* (Col. Chrys.) sur la plage de Banyuls-sur-Mer (Pyrénées Orientales). Bulletin de la Société Entomologique de France 55(8): 119–120.

67. Contribution à l’étude des *Microtheca* Stal (Col. Chrys.) (1re note). Bulletin de l’Institut Royal des Sciences Naturelles de Belgique 26(48): 1–27.

68. Rectifications de nomenclature chez les Chrysomelidae (1re note). Bulletin de l’Institut Royal des Sciences Naturelles de Belgique 26(56): 1–4.

**1951**

69. Contribution à l’étude du genre *Gastrophysa* Chevrolat (Col. Chrys.) (1re note). Bulletin de l’Institut Royal des Sciences Naturelles de Belgique 27(9): 1–11.

70. Quelques points de nomenclature restés longtemps litigieux chez les Chrysomélides. L’Entomologiste 7(1): 36–37.

71. Contribution à l’étude du genre *Gastrophysa* Chevrolat (Col. Chrys.) (2me note). Bulletin de l’Institut Royal des Sciences Naturelles de Belgique 27(13): 1–12.

72. Un nouveau *Chlamys* du Congo Belge (Col. Chlamisidae). Revue de Zoologie et de Botanique Africaines 44(3): 247–250.

73. Rectifications à propos de la notice sur V. Laboissière. Miscellanea Entomologica 46(1): 2.

74. Contribution à l’étude du genre *Gastrophysa* Chevrolat (Col. Chrys.). (3me note). Bulletin de l’Institut Royal des Sciences Naturelles de Belgique 27(21): 1–47.

75. Les parasites des Coléoptères français (5me liste). Miscellanea Entomologica 46(1): 3–5.

76. Contribution à l’étude des *Timarcha* (XVII) - Biologie. Miscellanea Entomologica 46(1): 6.

77. Pariades anormales chez les Chrysomélides (Col.). Miscellanea Entomologica 46(1): 7.

78. Contribution à l’étude des *Microtheca* Stal (Col. Chrys.). (2me note). Bulletin de l’Institut Royal des Sciences Naturelles de Belgique 27(38): 1–7.

79. P. Jolivet &Théodoridès, J. Les parasites, phorétiques et prédateurs des Chrysomeloidea (Col.) (2me note). Bulletin de l’Institut Royal des Sciences Naturelles de Belgique 27(25): 1–55.

80. Les Chrysomélides rares de la Belgique I. *Timarcha metallica* Laich. Naturalistes Belges 32(9–10): 150–155.

81. Variations géographiques chez deux espèces du genre *Chrysolina* Motschulsky Bulletin de l’Institut Royal des Sciences Naturelles de Belgique 27(51): 1–7.

82. Contribution à l’étude des Coléoptères Chrysomeloidea de la région Indo-Australienne (1° note). Bulletin de l’Institut Royal des Sciences Naturelles de Belgique 27(52): 1–7.

83. Notes sur la faune des Hautes Fagnes en Belgique. Bulletin et Annales de la Société Royale Entomologique de Belgique 87(7–8): 151–168.

84. Champignons entomophytes parasites de myrmécophiles. Bulletin et Annales de la Société Royale Entomologique de Belgique 87(9–10): 194.

85. Rectifications de nomenclature. Bulletin et Annales de la Société Royale Entomologique de Belgique 87(9–10): 194.

86. Un nouveau *Stenoplatys* Baly du Congo belge (Col. Galerucidae). Bulletin de l’Institut Royal des Sciences Naturelles de Belgique 27(58): 1–4.

87. Jolivet P, Théodoridès, J.: Capture accidentelle d’une Casside exotique à Banyuls. Vie et Milieu 2(2): 200.

88 Jolivet P, Théodoridès, J.: A propos de la plante-hôte de *Timarcha interstitialis* Fairmaire. Vie et Milieu 2(2): 281.

89. Megalopodidae (Col. Chrys.). Exploration du Parc National de l’Upemba, Mission G.F. De Witte 4(2): 13–14.

90. Sagridae (Col. Chrys.). Exploration du Parc National de l’Upemba, Mission G.F. De Witte 4(3): 15.

**1952**

91. Les Chrysomèles rares de la Belgique II. *Chrysolina purpurascens* Germ. Naturalistes Belges 35(1–2): 23–28.

92. Jolivet P, Jolivet, Y.: Contribution à l’étude des Coléoptères Chrysomeloidea de la région Indo-Australienne (2me note). Bulletin de l’Institut Royal des Sciences Naturelles de Belgique 28(5): 1–7.

93. Quelques données sur la myrmécophilie des Clytrides (Col. Chrys.). Bulletin de l’Institut Royal des Sciences Naturelles de Belgique 28(8): 1–12.

94. Sagridae (Col. Chrys.). Exploration du Parc National Albert, Mission G.F. De Witte 77(4): 31–32.

95. Clytridae (Col. Chrys.). Exploration du Parc National Albert, Mission G.F. De Witte 77(5): 33–43.

96. Les *Cyrtonus* de France (Col. Chrys.). L’Entomologiste 7(4–5): 106–109.

97. Allotrophie et Chrysomélides. Naturalistes Belges 33(3–4): 53–59.

98. *Timarcha* et folklore. Bulletin de la Société Linnéenne de Lyon 21(3): 79–80.

99. La loi de Maulik et les plantes-hôtes des Chrysomélides. L’Entomologiste 7(6): 154–156.

100. Jolivet P, Théodoridès, J.: Les parasites, phorétiques et prédateurs des Chrysomeloidea (Col.). (3me note). Bulletin de l’Institut Royal des Sciences Naturelles de Belgique 28(20): 1–19).

101. Les Chrysomèles rares de la Belgique III. Les Haemonia. Naturalistes Belges 33(5–6): 92–97.

102. Contribution à l’étude des Coléoptères Chrysoméloidea de la région Indo-Australienne. 3me note). Bulletin de l’Institut Royal des Sciences Naturelles de Belgique 28(28): 1–4.

103. A propos d’un endophyte de *Chrysocarabus auronitens* F. Bulletin et Annales de la Société Royale Entomologique de Belgique 88(3–4): 67–68.

104. A propos de l’écologie de *Chrysolina menthastri* Suffr. (Col. Chrys.). Vie et Milieu 2(4): 470–472.

105. A propos des modifications dans le rythme des métamorphoses des Coléoptères. Vie et Milieu 2(4): 530–531.

106. Captures de Tiques à Majorque. Vie et Milieu 2(4): 530.

107. Un *Hydrothassa* nouveau pour la faune française. Vie et Milieu 2(4): 532.

108. Jolivet P, Théodoridès, J.: Capture de *Chrysolina* (Col. Chrys.) du Midi de la France. Vie et Milieu 2(4): 533.

109. Captures de Myrmécophiles. Bulletin et Annales de la Société Royale Entomologique de Belgique 88(5–6): 117.

110. Remarques préliminaires sur l’écologie de *Timarcha balearica* Gory (Col. Chrys.). Bulletin et Annales de la Société Royale Entomologique de Belgique 88(5–6): 138–140.

111. Au sujet du terme *Chrysolina*. L’Entomologiste 8(2): 43–44.

112. Chrysomelidae (Col. Chrys.). Exploration du Parc National de l’Upemba, Mission G.F. De Witte 8(3): 23–27.

113. Notes biométriques sur quelques Chrysomeloidea (Col.). Bulletin de l’Institut Royal des Sciences Naturelles de Belgique 28(44): 1–8.

114. Quelques données complémentaires sur l’allotrophie chez les Chrysomélides. Naturalistes. Belges 33(9–12): 135–138.

115. Uhmann E, Jolivet, P.: Sur deux espèces d’Hispinae de la collection Chapuis. Bulletin de l’Institut Royal des Sciences Naturelles de Belgique 28(61): 1–8).

**1953**

116. A propos des parasites et phorétiques de *Timarcha nicaaensis* Villa. Bulletin et Annales de la Société Royale Entomologique de Belgique 89(3–4): 67–68.

117. A propos de la plante-hôte de *Timarcha punctella* Marseul. Bulletin et Annales de la Société Royale Entomologique de Belgique 89(5–6): 117–118.

118. Jolivet P, Théodoridès, J.: Les parasites, phorétiques et prédateurs des Chrysomeloidea (Col.) (4me note). Bulletin de l’Institut Royal des Sciences Naturelles de Belgique 29(32): 1–5).

119.-Les Chrysomeloidea (Col.) des Iles Baléares. Memoires de l‘Institut Royal des Sciences Naturelles de Belgique 2(50):1–88.

120. De la nomenclature du genre *Chlamys* Knoch (Col.) Revue de Zoologie et de Botanique Africaines 47(3–4): 258–260.

121. Contribution à l’ étude de la zone d’inondation du Niger (mission G. Remaudière,1950) IV. Col. Chrysomeloidea. Bulletin de l’Institut Fondamental d’Afrique Noire 15(4): 1539–1547.

122. Jolivet P, Théodoridès, J.: Sur une fourmi prédatrice d’une larve de Chrysomélide. Vie et Milieu 4(1): 131).

**1954**

123. Notes écologiques préliminaires sur les Chrysomeloidea de la Sierra Nevada. Bulletin et Annales de la Société Royale Entomologique de Belgique 90(3–4): 69–72.

124. Notes écologiques préliminaires sur les Chrysomeloidea des Iles Canaries. Bulletin et Annales de la Société Royale Entomologique de Belgique 90(3–4): 73- 82.

125. Arthropodes récoltés à Cabrera. Vie et Milieu 4(3): 553–555.

126. Phytophagie et Sélection trophique. Institut Royal des Sciences Naturelles de Belgique, Bruxelles: Livre Jubilaire Victor Van Straelen 2: 1099–1134.

127. Clytrides nouveaux ou rares de l’Afrique Centrale (Col. Chrys.). Miscellanea Zoologica H. Schouteden. Annales du Musee Royal du Congo Belge. Nouvelle serie in-4°, Sciences zoologiques, volume 1: 415–418.

128. Un nouveau champignon entomophage en Belgique. Bulletin et Annales de la Société Royale Entomologique de Belgique 90(5–6): 121.

129. Nouveaux Chrysomélides parasités. Bulletin et Annales de la Société Royale Entomologique de Belgique 90(5–6): 121–122.

130. Cryptocephalidae (Col. Chrys.). Exploration du Parc National de l’Upemba, Mission G.F. De Witte 25(5): 97–112.

131. Jolivet P, Théodoridès, J.: Les parasites, phorétiques et prédateurs des Chrysomeloidea (Col.). (5me note). Bulletin de l’Institut Royal des Sciences Naturelles de Belgique 30(23): 1–12).

132. Nouvel accouplement interspécifique chez *Chrysolina*. Bulletin et Annales de la Société Royale Entomologique de Belgique 90(9–10): 233–234.

133. Mission E. Janssens et R. Tiollet en Grèce (3me note). Col. Chrysomeloidea. Bulletin et Annales de la Société Royale Entomologique de Belgique 90(9–10): 287–289.

134. L’aile des Chrysomeloidea (Col.). Miscellanea Entomologica 48: 85–101.

135. Notes biogéographiques sur les Chrysomeloidea des Iles Baléares. Miscellanea Entomologica 48: 102–105.

136. Jolivet P, Théodoridès, J.: Présence de *Pteromalus puparum* L. aux îles Canaries. Vie et Milieu 5(3): 458).

137. Contribution à l’étude de la faune entomologique du Ruanda-Urundi. Mission P. Basilewsky 1953. 63. Col. ChrysomelidaeCryptocephalinae. Annales du Musee Royal du Congo Belge, Sciences Zoologiques 40: 200–203.

138. Recherches sur les organes facettiques des ailes des insectes (1). Bulletin de l’Institut Royal des Sciences Naturelles de Belgique 31(17): 1–23.

139. Clytridae (Col. Chrysomeloidea). Exploration du Parc National de l’Upemba, Mission G.F. De Witte 35(4): 63–81.

140. Crioceridae (Col. Chrysomeloidea. Exploration du Parc National de l’Upemba, Mission G.F. De Witte 38(4): 51–83.

**1956**

141. Présence de *Gregarina munieri* (A. Schneider, 1876) aux îles Canaries. Bulletin et Annales de la Société Royale Entomologique de Belgique 92(5–6): 102–103.

**1957**

142. Familia Chrysomelidae Subfamilia Orsodacninae. Pp. 1–16 in Hincks, W.D. (ed.) Coleopterorum Catalogus Supplementa 51(3). W. Junk, Amsterdam.

143. de Zuleta, J.; Jolivet, P.; Thymakis K, Caprari, P.: Seasonal variations in susceptibility to DDT of *Anopheles maculipennis* in Iran. Bulletin of the World Health Organisation 16(2): 475–479.

144. de Zulueta, J.; Jolivet, P.; Thymakis K, Caprari, P.: A survey of Anopheline susceptibility to insecticides in Iran. Report prepared by the WHO Malaria Advisory Team No. 2 WHO/Mal/188: 1–18 (English & French version).

145. de Zulueta, J.; Jolivet, P.; Thymakis K, Caprari, P.: Report prepared by the WHO Malaria Advisory Team N°2 in Iran, 4 July-1 Nov. 1956, WHO/AS/33.57. 26 March 1957: 1–32, 8 appendices).

146. Farinaud, M.E.; Jolivet, P.; Thymakis K, Caprari, P.: Report of Advisory Team on Malaria Eradication N° 4 in Taiwan, China, January - April 1957, WHO/AS/103.57, July 1957: 1–68, 3 annexes).

147. Report on the Entomological Survey in Taiwan January - April 1957. WHO Malaria Advisory Team N°4: 25 pp.

148. Gregoire, Ch. & Jolivet, P.: Coagulation du sang chez les Arthropodes. Exploration du Parc National Albert 2me série 2(4): 1–45, 9 pls.

149. Tuzet, O.; Manier, J.-F.; & Jolivet, P.: Nyctotherus parasites de l’intestin de Myriapodes. Exploration du Parc National Albert 2me série 2(5): 7–19.

150. Tuzet, O.; Manier, J.-F.; & Jolivet, P.: Trichomycètes monoaxes et rameux de l’intestin postérieur de Polydesmida, Spirostreptida et Spirobolida. Exploration du Parc National Albert 2me série 5(3): 21–38, 8 pls.

151. Tuzet, O.; Manier, J.-F.; & Jolivet, P.: Grégarines parasites de Myriapodes Diplopodes. Exploration du Parc National Albert 2me série 5(4): 57–76).

152. ColeopteraChrysomeloidea. Institut Royal des Sciences Naturelles de Belgique, Exploration Hydrobiologique du Lac Tanganika (1946–1947), Résultats Scientifiques 3(6): 46–51.

153. Recherches sur l’aile des Chrysomeloidea (Coleoptera). 1re partie. Memoires de l’Institut Royal des Sciences Naturelles de Belgique 2(51): 1–180, 20 pls.

**1958**

154. Théodoridès, J.; Ormières R, Jolivet, P.: Eugrégarines parasites d’Orthoptéroides. Exploration du Parc National Albert 2me série 7: 1–44, 6 pls., 1 map.

**1959**

155. Théodoridès J, Jolivet, P.: Eugrégarines, parasites de Coléoptères. Exploration du Parc National Albert 2me série 8: 1–95, 5 pls., 1 map.

156. Jeekel, C.A.W.; Tuzet, O.; Manier, J. F. & Jolivet, P.: Myriapodes et leurs parasites. Exploration du Parc National Albert 2me série 9(1): 3–32, 4 pls., 1 map.

157. Observations on the Ethiopian Anopheline Mosquitoes and their susceptibility to insecticides. Report on the 2nd Regional Conference on Malaria Eradication, Addis Abeba, 16–21 November 1959. EM/ME-Tech. 2/23: 1–15.

158. Recherches sur l’Aile des Chrysomeloidea 2me partie. Memoires de l’Institut Royal des Sciences Naturelles de Belgique 2(58): 1–152, 20 pls.

**1961**

159. Nouveaux hôtes et nouvelles localités de capture de Gregarina crenata (Bhatia & Setna) (Gregarinidae). Bulletin et Annales de la Société Royale Entomologique de Belgique 97(7–8): 237–238.

**1963**

160. Théodoridès J, Jolivet, P.: Quelques Grégarines Stylocéphalides parasites de Coléoptères Ténébrionides d’Egypte. Annales de la Parasitologie Humaine et Comparée 38(1): 11–21).

161. Rapport général sur l’Enseignement scientifique en Algérie. Rapports UNESCO, ED/462/1205, Sept. 1963).

**1964**

162. Théodoridès J, Jolivet, P.: Grégarines parasites de Coléoptères d’Ethiopie. Annales de la Parasitologie Humaine et Comparée 39(1): 1–31).

163. Programmes et Enseignement des Sciences Naturelles dans les Ecoles Normales Supérieures. UNESCO, Réunion des Directeurs et Professeurs de Sciences des ENS d’Afrique, Abidjan 2–12 Juillet 1964. UNESCO Reg. Cent. Ed. Inf. Africa, Accra Occasional paper 3: 12–26.

164. Serie, C; Andral, L.; Lindrec, A.; Poikier, A.; Jolivet P, Neri, P.: La fièvre jaune en Ethiopie. Annales de l’Institut Pasteur d’Ethiopie, Addis Abeba 2(5): 1–179.

**1965**

165. Théodoridès, J.; Desportes, I.. & Jolivet, P.: Grégarines parasites de Coléoptères Ténébrionides de la région de Khartoum, République du Soudan. Bulletin de l’Institut Fondamental d’Afrique Noire 27(A 1): 139–164.

166. Refresher course for experts in scientific disciplines. Dar-ès-Salam. UNESCO/SF/ENS Africa 15, Paris: 1–8.

167. Note sur l’écologie des *Timarcha* marocaines (Col. Chrys.). Bulletin de la Société des Sciences naturelles et physiques du Maroc 45(3–4): 159–190.

**1966**

168. Une nouvelle sous-espèce de *Timarcha* marocaine (Col. Chrys.). Bulletin de la Société des Sciences naturelles et physiques du Maroc 46(1–2): 121–126.

169. Notes systématiques et écologiques sur les Chrysomélides marocains (Col.) (1re note: Chrysomelinae). Bulletin de la Société des Sciences naturelles et physiques du Maroc 46(1–2): 121–126.

170. Plantes-hôtes de quelques Chrysomélides d’Afrique centrale. Bulletin et Annales de la Société Royale Entomologique de Belgique 102: 59–60.

171. Sur une pariade anormale chez deux espèces de *Timarcha* (Col. Chrys.). Bulletin de la Société des Sciences Naturelles et Physiques du Maroc 46: 10–12.

172. Sur une migration de *Vanessa cardui* L. (Lep. Nymphalidae) au Maroc. Bulletin de la Société des Sciences Naturelles et Physiques du Maroc 46: 12–13.

**1967**

173. Nouvelles observations écologiques chez les *Timarcha* Latr. (Col. Chrys.). Bulletin de la Société Linnéenne Lyon 36(1): 13–18.

174. Sur la position systématique des genres *Xenomela* Weise et *Oreomela* Jacobson (Col. Chrys.). Bulletin de la Société Linnéenne Lyon 36(7): 309–316.

175. Notes systématiques et écologiques sur les Chrysomélides marocains (Col.) (2me note). Bulletin de la Société des Sciences Naturelles et Physiques du Maroc 46(3–4): 305–393.

176. Curieuses modifications de l’instinct chez les abeilles africaines. Bulletin de la Société des Sciences Naturelles et Physiques du Maroc 46(5–8): 178.

177. Les races de *Timarcha punctella* Latreille (Col. Chrys.) au Maroc. Bulletin de la Société Linnéenne Lyon 37(2): 92–95 & 37(3): 113–118.

178. De la position systématique du genre *Microdonacia* Blackburn (Col. Chrys.). Entomologische Arbeiten aus dem Museum Georg Frey 19: 188–192.

179. Notes écologiques sur les *Timarcha* tuniso-lybiens (Col. Chrys.). Bulletin de la Société Entomologique de France 72(9–10): 224–239.

**1968**

180. Le complexe de *Timarcha scabripennis* Fairmaire au Maroc (Col. Chrys.). Entomologische Arbeiten aus dem Museum Georg Frey 19: 229–236.

181. Les Alticides vénéneux de l’Afrique du Sud. L’Entomologiste 23(4): 100–111.

182. Les Chrysomelidae (Col.) du Massif du Debdou (Maroc). Eos, Revista Española de Entomologia 43(3–4): 565–573.

183. Capture en abondance au Maroc de l’espèce *Timarcha tingitana* Fairmaire (Col. Chrys.). Bulletin et Annales de la Société Royale Entomologique de Belgique 103: 329–330.

184. Les Donaciinae de l’Afrique du Nord (Col. Chrys.). Bulletin de la Société Linnéenne Lyon 37(7): 307–315.

**1970**

185. Familia Chrysomelidae Subfamilia Donaciinae. In Steel, W.O. (Ed.) Coleopterorum Catalogus Supplementa 51(2): 1–71.

186. A propos de l’extrême toxicité d’une espèce géante de Myriapode Diplopode de la Nouvelle-Guinée Australienne appartenant au genre *Polyconoceras* Attems (Rhinocricidae). L’Entomologiste 26(1–2): 31–40 & 26(3): 47–54.

**1971**

187. Sélection trophique et adaptation écologique chez le genre *Timarcha* Latreille (Col. Chrys.). Proceedings of the 13th International Congress of Entomology, Moscow 1968, 1: 505–506.

188. La Nouvelle-Guinée Australienne. Introduction écologique et entomologique. Cahiers du Pacifique 15: 41–70.

189. Une extraordinaire concentration de Ténébrionides dans l’arboretum de Phu Kae, près Sara Buri, Thailande. Bulletin et Annales de la Société Royale Entomologique de Belgique 106(10–11): 323–326.

190. Quelques remarques à propos de la plante-hôte des *Platycorinus* Chevrolat en Thailande (Col. Chrys. Eumolpinae). Bulletin de la Société Linnéenne Lyon 40(7): 189–195.

191. A propos des Insectes à boissons et des insectes à sauce. L’Entomologiste 27(1–2): 3–5.

192. La toxicité des Myriapodes du Sud-Est Asiatique. L’Entomologiste 27 6): 156–158.

193. A propos de la plante-hôte de la Chrysomèle des Orchidées: Lema (Petauristes) pectoralis Baly, 1865, en Thailande, Malaisie et Bornéo (Col. Chrys.). Bulletin de la Société Entomologique de France 76(11–12): 248–252.

194. Rectifications de nomenclature chez les Chrysomélides: le genre *Procris*. Bulletin et Annales de la Société Royale Entomologique de Belgique 107(10–11): 382.

**1972**

195. ColeopteraChrysomelidaeDonaciinae. Exploration du Parc National de la Garamba, Mission H. de Saeger 56(3): 43–58.

196. Théodoridès, J.; Desportes I, Jolivet, P.: Grégarines de Nouvelle-Guinée et des îles voisines. Cahiers du Pacifique 16: 109–168.

197. Recherches sur *Nisotra gemella* (Erichson, 1834), l’Altise du kenaf (*Hibiscus sabdariffa* L.) en Thailande. Sélection trophique du complexe *Nisotra*/*Podagrica* (Col. Chrys. Alticinae). Bulletin de la Société Linnéenne Lyon 41(7): 138–148.

198. Le problème des Chrironomides (Dipt. Némat.) dans le Languedoc-Roussilllon. Note préliminaire. Vie et Milieu 23(2): 269–290.

199. An Interpretation of the Host Plants selected by Species of the genus *Timarcha* (Col. Chrys.). Korean Journal of Entomology 12(1): 21–26.

200. Observations sur les plantes-hôtes de *Timarcha* du Midi de la France (Col. Chrys.). Annales de la Société d’Horticulture et d’Histoire Naturelle de l’Hêrault, Montpellier 112 (4): 330–332.

201. A propos des *Donacia* de la Thaïlande (Col. Chrys. Donaciinae. Bulletin de la Société Linnéenne Lyon 41(9–10): 193–201.

202. Quelques réflexions sur les perspectives actuelles de la lutte biologique contre les moustiques (Dipt. Culicidae). Vie et Milieu 23(2): 357–366.

**1973**

203. Jolivet P, Petitpierre, E.: Plantes-hôtes connues de *Timarcha* Latreille (Col. Chrys.). Quelques considérations sur les raisons possibles du trophisme sélectif. Bulletin de la Société Entomologique de France 78(1–2): 9–25.

204. Les Plantes Myrmécophiles du Sud-Est Asiatique. Cahiers du Pacifique 17: 41–69.

205. ULV application of Fenitrothion from a large aerosol generator for the control of *Culex tritaeniorhynchus*. WHO/VBC/73, Geneva 424: 1–9.

206. Self, L.S.; Shim, J.C. & Jolivet, P.: Susceptibility of *Culex tritaeniorrhynchus* and six other mosquitoes to insecticides in Korea,1969 to 1972. WHO,VBC/73, Geneva 425: 1–6.

207. Some considerations about the actual prospects of the biological control against mosquitoes (Dipt. Culicidae). Korean Journal of Entomology 3(1):1–8.

**1974**

208. Essai d’analyse écologique de la faune chrysomélidienne de la Corée (Col. Chrys.). Cahiers du Pacifique 17: 253–288.

209. Réflexions sur la distribution du genre *Gastrophysa* Chevrolat, 1837, en Corée méridionale (Col. Chrys. Chrys.). Bulletin de la Société Entomologique de France 79 (1–2): 3–6.

211. Jolivet, P.; Yi, B.-G.; Ree, H.-I.; Lee, K.-W.: A trial for application of Phytoecological Cartography to detect the Mosquito breeding places on an island of the Yellow Sea, Korea. Korean Journal of Entomology 4(1): 15–29.

212. Les Chrysomèles des Araliacées (Col.). Bulletin de la Société Linnéenne Lyon 43(4): 113–120.

213. Jolivet P, Ree, H.-I.: Arthropods of Public Health Importance in Korea (Supplementary List). Korean Journal of Entomology 4(1): 10–14).

214. Self, L.S.; Shim, J.C. & Jolivet, P.: Susceptibility of *Culex tritaeniorhynchus* and six other mosquitoes to insecticides in Korea. Cahiers de l’ Office de la Recherche Scientifique et Technique Outre-Mer série Entomologie Médicale & Parasitologie 12 (2): 81–92.

215. Jolivet, P.; Yi, B.-G.; Ree, H.-I. & Lee, K.-W. Application of Phytoecological cartography to detect the mosquito breeding places on an island of the Yellow Sea, Korea. WHO/VBC/74.485, Geneva: 1–14.

216. Mathis, H.L., Shim, J.C, Ree, H.-I. & Jolivet, P.: A Helicopter application of ULV Dibrom against *Culex tritaeniorhynchus* in Seoul, Korea. WHO/VBC/74.501, Geneva: 1–6.

217. Mathis, H.L. & Jolivet, P.: Seasonal and yearly population variations of adult *Culex tritaeniorhynchus* Giles, in Korea. WHO/VBC/74.499, Geneva: 1–7).

218. Rectifications and additions to my list of Korean Chrysomelidae (Col.) Korean Journal of Entomology 4(2): 97–99.

**1975**

219. Une excursion entomologique à l’île de Quelpart (Cheju-Dô) et découverte d’une espèce alpine nouvelle pour la Corée de *Chrysolina* Motschulsky (Col. Chrys.). Réflexions sur la plantagophagie. Bulletin de la Société Linnéenne Lyon 44(2): 57–64 & 44 (3):72–81.

220. Théodoridès, J.; Desportes I, Jolivet, P. Grégarines de la Thaïlande. Annales de Parasitologie Humaine et Comparée 50(2): 145–159.

221. Mathis, H.L.; Ree, H.I.; Jolivet P, Shim, J.C.: A field trial of the Insect growth inhibitors, OMS-1697 (Altosid) and OMS-1804 against *Culex pipiens* in Seoul, Korea. WHO/VBC/75.494, Geneva 1–5.

222. Jolivet, P.; Self, L.S & Wada, Y.: Studies on the over-wintering of *Culex tritaeniorhynchus* Giles, 1901 in the republic of Korea. WHO/VBC/75.514, Geneva: 1–5.

223. Jolivet, P.; Ree, H.I.; Hong, H.K. & Wada, Y.: Dispersal of *Culex tritaeniorhynchus* in Korea. WHO/VBC/75.599, 1–6.

224. Arata A, Jolivet, P.: La Fièvre Jaune. WHO, Geneva & Lomé, SEM/ANT. Larv. /75/10: 1–12.

225. Arata. A. & Jolivet, P.: Le contrôle biologique des Vecteurs: Situation actuelle et perspectives d’avenir. WHO, Geneva & Lomé, SEM/ANT. Larv. /75/20: 1–7.

226. Vector Control Research Unit (VECRU). Final Report. 1969–1974. VBC/75.3. Geneva 1- 25.

**1976**

227. Théodoridès, J.; Desportes, I.& Jolivet, P.: Grégarines de la Corée du Sud. Annales de Parasitologie Humaine et Comparée 51(2): 161–173.

228. Notes préliminaires sur la biologie des *Timarcha* du Pacifique Nord- Occidental Américain (*Americanotimarcha* Jolivet) (Col. Chrys.). Cahiers du Pacifique 19: 153–165.

229. Jolivet, P.; Yi, B.-G.; Ree, H.-I & Lee, K.-W.: Essai d’application de la cartographie phytoécologique à la détection des gîtes larvaires des moustiques dans une île de la Mer Jaune, en Corée. Cahiers du Pacifique 19: 113–132.

230. Jolivet P, Petitpierre, E.: Les plantes-hôtes connues des *Chrysolina* (Col. Chrys.). Essai sur les types de sélection trophique. Annales de la Société Entomologiqe de France (N.S.) 12(1): 123–149.

231. Jolivet P, Petitpierre, E.: Sélection trophique et évolution chromosomique chez les Chrysomelinae (Col. Chrysomelidae). Acta Zoologica et Pathologica Antverpiensia 66: 59–90.

232. Petitpierre E, Jolivet, P.: Phylogenetic Position of the American *Timarcha* Latr. (Coleoptera, Chrysomelidae) based on Chromosomal Data. Experientia 32(2): 157–158.

233. Ree, H.-I.; Wada, Y.; Jolivet, P.; Hong, H.K.; Self, L.S. & Lee, K.-W.: Studies on over-wintering of *Culex tritaeniorhynchus* Giles in the Republic of Korea. Cahiers de l’ Office de la Recherche Scientifique et Technique Outre-Mer série Entomologie Médicale & Parasitologie 14(2): 105–109.

**1977**

234. Jolivet P, Van Parys, E.: Un cas inédit de mimétisme agressif entre un Chrysomélide (*Mesoplatys cincta* Olivier) et un Carabique (*Cyaneodinodes ammon* Fabricius) (Col.). Bulletin de la Société Linnéenne Lyon 46(6): 168–180.

235. Jolivet, P.; Hong, H.K.; Lee, C.S. & Mathis, H.L.: Ground aerosol of Ultra-Low-Volume Dibrom against *Culex tritaeniorhynchus* in Pusan, Korea. Korean Journal of Entomology 7(2): 29–32.

236. Sélection trophique chez les *Eupoda* (Col. Chrys.). Bulletin de la Société Linnéenne Lyon 46(9): 321–336.

237. Ouattara, S.; Jolivet P, Van Parys, E.: Première et Seconde liste des Insectes et des Plantes-hôtes en Haute-Volta et dans les pays limitrophes. Projet de Renforcement de la Protection des Plantes en Haute-Volta, Bobo-Dioulasso (Upper Volta) Nov. 1976 & Avril 1977: 93 & 107 pp., mimeographed.

**1978**

238. Sélection trophique chez les Clytrinae, Cryptocephalinae et Chlamisinae (Camptosoma) et les Lamprosomatinae (Cyclica) (Coleoptera, Chrysomelidae). Acta Zoologica et Pathologica Antverpiensia 70: 167–200.

**1979**.

239. Les Chrysomelidae (Col.) des *Citrus* et apparentés (Rutaceae) en zone tempérée et tropicale. Bulletin de la Société Linnéenne Lyon 48(4): 197–200 & 249–256.

240. Réflexions sur l’écologie, l’origine et la distribution des Chrysomélides (Col.) des Iles Mascareignes, Océan Indien, avec la description de deux espèces nouvelles. Bulletin de la Société Linnéenne Lyon 48(9): 524–528 & 48(10): 606–608, 641–649.

**1980**

241. Les Insectes et l’Homme. 128 pp., Presses Universitaires de France, Paris.

242. Desportes, I.; Jolivet, P.; Mateu J, Thédoridès, J.: Présence de *Gregarina ophoni* Tuzet et Ormières,1956 (Eugreg. Gregarinidae) chez Notiobia (Anisotarsus) bradytoides (Bates) (Col. Carabidae) en Equateur. Annales de Parasitologie Humaine et Comparée 55(1): 145–146.

243. Les Mannes: Entomologie et Botanique. Bulletin de la Société Linnéenne Lyon 49(9): 17–22.

**1981**

244. Jolivet P, Petitpierre, E.: Biology of Chrysomelidae (Coleoptera). Butlletí de la Institució Catalana d’Història Natural (Secció de Zoologia 4) 47: 105–138.

245. Bourdouxhe L, Jolivet, P.: Nouvelles observations sur le complexe mimétique de *Mesoplatys cincta* Olivier (Col. Chrysomelidae) au Sénégal. Bulletin de la Société Linnéenne Lyon 50(2): 46–48).

246. Théodoridès J, Jolivet, P.: *Anisolobus desportis* n. sp. (EugregarinaGregarinidae) parasite d’un Coléoptère Curculionide de La Réunion. Considérations sur le genre *Anisolobus* Vincent. Annales de Parasitologie Humaine et Comparée 56(5): 461–466).

**1982**

247. Théodoridès J, Jolivet, P.: Sur la présence de *Stylocephalus africanus* Théod., Desp. & Jolivet, 1965 (Eugreg. Stylocephalidae) chez un *Zophosis* (Col. Ténebrionidae) au Sénégal. Annales de Parasitologie Humaine et Comparée 57(1): 108).

248. Les Eumolpinae (Col. Chrys.) des Apocynaceae et des Asclepiadaceae (Gentianales.Bulletin de la Société Linnéenne Lyon 51(7): 214–222.

**1983**

249. Theodorides, J.; Ormieres R, Jolivet, P.: *Gregarina labidurae* n. sp. parasite de *Labidura riparia* Pallas. Remarques sur les Grégarina de Dermaptères. Vie et Milieu 32(1): 73–75).

250. Les Insectes et les Plantes. Evolution parallèle et Adaptations. Bulletin de la Société Linnéenne Lyon 52: 1–146.

251. Théodoridès, J.; Jolivet P, Desportes, I.: Grégarines d’Arthropodes du Nord-Vietnam. Annales de Sciences Naturelles Zoologie 13e série 6: 57–69.

252. Un hémimyrmécophyte à Chrysomélides (Col.) du Sud-Est Asiatique, *Clerodendrum fragrans* (Vent.) Willd. (Verbenaceae). Bulletin de la Société Linnéenne Lyon 52(8): 242–261.

**1984**

253. *Phaedon fulvescens* Weise (Col. Chrys.). Un auxiliaire possible dans le contrôle des *Rubus* aux Tropiques. Bulletin de la Société Linnéenne Lyon 53(7): 235–246.

**1985**

254. Théodoridès J, Jolivet, P.: Eugrégarines d’Arthropodes terrestres des Iles du Cap Vert. Anais da Faculdade de Ciências da Universidade do Porto 67(1–4): 5–33).

255. Un myrmécophyte hors de son pays d’origine: *Clerodendrum fallax* Lindley, 1844 (Verbenaceae) aux Iles du Cap Vert. Bulletin de la Société Linnéenne Lyon 54(5): 122–128.

256. *Phaedon fulvescens*: A possible control agent of *Rubus* in the Tropics. Entomography 3: 499–500 (XVII International Congress of Entomology, Hamburg 1984, Abstracts: 934).

257. Comments on the Chrysomelidae of the Cape Verde Islands. Entomography 3: 502.

**1986**

258. Les découvertes récentes en myrmécophilie des Plantes. Tendances actuelles et interprétations. Pp. 41–52 in Jean-Henri Fabre: Anniversaire du Jubilé, 1910–1985, Actes du Congrès, Paris et le Vaucluse, 13–18 mai 1985. Le Léopard d’Or, Paris.

259. Le Millepatte de Santo Antao (Iles du Cap Vert) ou comment une espèce inoffensive peut devenir un ravageur! L’Entomologiste 42(1): 45–56.

260. Pierre Jolivet, Eduard Petitpierre & Mauro Daccordi. Les plantes-hôtes des Chrysomelidae. Quelques nouvelles précisions et additions (Coleoptera). Nouvelle Revue d’Entomologie (N.S.) 3(3): 341–357).

261. Insects and Plants. Parallel Evolution and Adaptations. 197 pp., E.J. Brill, New York (2nd. ed., Sandhill Crane Press, Gainesville, Florida 1992; 3rd. ed., St. Lucie Press, Boca Raton, Florida 1996).

262. Les Fourmis et les Plantes: Un exemple de coévolution. 254 pp., Fondation Singer-Polignac, Paris.

263. Réflexions sur la sélection trophique des Megascelinae et des Eumolpinae (Cyclica) (ColeopteraChrysomelidae). 2me Conférence Internationale des Entomologistes d‘Expression Française, Trois Rivières: 33.

264. Les Fourmis et la Télévision. L’Entomologiste 42(6): 321–323.

**1987**

265. Sélection trophique chez les Megascelinae et les Eumolpinae (Cyclica) (ColeopteraChrysomelidae). Bulletin de la Société Linnéenne Lyon 56(6): 199–208 & 56(7): 217–240.

266. Jan Bechyne: Des Souvenirs de Jeunesse. Boletim do Museo Paraense Emilio Goeldi, Serie Zoologia 3(2): 257–259.

267. Nouvelles Observations sur les Plantes à Fourmis. Réflexions sur la Myrmécophilie. L’Entomologiste 43(1): 39–52.

268. Remarques sur la biocénose des *Cecropia* (Cecropiaceae). Biologie des *Coelomera* Chevrolat avec la description d’une nouvelle espèce du Brésil (ColeopteraChrysomelidaeGalerucinae. Bulletin de la Société Linnéenne Lyon 56(8): 255–276.

269. Aperçu de la sélection trophique chez les Galerucinae. Etude genre par genre (ColeopteraChrysomelidae). Bulletin et Annales de la Société Royale Entomologique de Belgique 123(7–9): 283–307.

270. Premières données sur la biologie d’*Elytrosphaera xanthopyga* Stal (Col. Chrys.). Bulletin de la Société Entomologique de France 91(5–6): 163–170.

271. Les Plantes Carnivores. 126 pp., Collections: Sciences & Découvertes, Editions du Rocher, Paris.

272. Chapter 20: A case of parasitism of an Ant-*Cecropia* Mutualism in Brazil. Pp. 327–335 in Trager, J.C. (ed.) Advances in Myrmecology 1, Brill publishers, New York.

**1988**

273. Les Soins Parentaux chez les Chrysomelides. L’Entomologiste 44(2): 93–101.

274. Jolivet, P.; Petitpierre E, Hsiao, T. (Eds) Biology of Chrysomelidae. 613 pp., Kluwer Academic Publishers, Dordrecht.

275. Food Habits and Food Selection of Chrysomelidae. Bionomic and Evolutionary Perspectives. Pp. 1–24 in Biology of Chrysomelidae.

276. Vasconcellos-Neto J, Jolivet, P.: Une nouvelle stratégie de défense: la stratégie de défense annulaire (Cycloalexie) chez quelques larves de Chrysomélides brésiliens (Col.). Bulletin de la Société Entomologique de France 92(9–10): 291–299.

277. Sélection trophique chez les Cassidinae (ColeopteraChrysomelidae). Bulletin de la Société Linnéenne Lyon 57(9): 301–320.

278. Insectes et Plantes Carnivores. Courrier Nature, Paris 116: 22–28 & 117: 20–24.

279. A propos de la Myrmécophilie des Orchidées. L‘Orchidophile, Paris 84: 210–215.

280. Hawkeswood, T.J. & Jolivet, P.: Notes on the biology and behaviour of the Australian leaf beetle, *Cleptor inermis* Lefèvre, 1885 (ColeopteraChrysomelidaeEumolpinae). Bulletin et Annales de la Société Royale Entomologique de Belgique 124(3):189–194.

281. Ants, Leaf-beetles and Plants. P. 40 in: Annals of the International Symposium of the Evolutionary Ecology of Tropical Herbivores. Campinas State University, Campinas, Brazil.

282. An apterous beetle genus on Compositae: *Elytrosphaera*. P. 39 in: Annals of the International Symposium of the Evolutionary Ecology of Tropical Herbivores. Campinas State University, Campinas, Brazil.

**1989**

283. Sélection trophique chez les Hispinae (Col. Chrys.). Bulletin de la Société Linnéenne Lyon 58 (9): 297–317.

284. Ring defense strategy among Brazilian chrysomelid larvae (Coleoptera). Proceedings of the XVII International Congress of Entomology Vancouver: 36 & Entomography 6: 347–354.

285. The Chrysomelidae of *Cecropia* (Cecropiaceae): A strange cohabitation. Proceedings of the XVII International Congress of Entomology Vancouver: 37 & Entomography 6: 391–395.

286. A propos des *Timarcha* Nord-Américains (Col. Chrysomelidae). L’Entomologiste 45(1): 27–34.

287. Des succulentes épiphytes et myrmécophiles: *Myrmecodia* et *Hydnophyta* (Rubiacées) en Asie du Sud-Est. Succulentes, Monaco 2: 11–18.

288. Une stratégie originale: la défense en cercle des larves d’Insectes. La Recherche, Paris 20(211): 804–806.

289. Un genre en danger de mort: *Timarcha*. L’Entomologiste 45(6): 301–310.

**1990**

290. Théodoridès J, Jolivet, P.: Eugrégarines de Coléoptères du Brésil. Descriptions de trois espèces nouvelles et notes biologiques. Boletim do Museo Paraense Emilio Goeldi Zoologia 6(1): 47–58.

291. Fourmis, *Cecropia* et épiphytes. L’Entomologiste 46(2–3): 121–127.

292. Relative protection of *Cecropia* trees against Leaf-cutter ants in Tropical America. Pp. 251–254 in: Van der Meer, R.K.; Jaffe K, Cedeno, A. (Eds) Applied Myrmecology, Westview Press, Boulder 24: 251- 254.

293. A la Recherche du Monde Perdu: Les tépuys du Vénézuela. Courrier de la Nature, Paris 124: 32–35 & 125: 28–32.

294. Jolivet, P.; Vasconcellos-Neto J, Weinstein, Ph.: Cycloalexy: A new concept in the larval defense of Insects. Insecta Mundi 4(1–4): 133–142.

295. Distribution et Plantes-hôtes de *Chrysolina staphylea* (Linné, 1758). Bulletin et Annales de la Société Royale Entomologique de Belgique 126(7–9): 123–130.

296. La Dionée attrape-mouches en danger de mort. Dionée 18: 2–6.

297. Le genre *Ledothamnus* est-il carnivore? Dionée 20: 1–5.

**1991**

276. Sélection trophique chez les Alticinae (Col. Chrysomelidae). Bulletin de la Société Linnéenne Lyon 60(1): 26–40 & 60(2): 53–72.

298. Ants, Plants and Beetles. A triangular relationship. Chapter 26, pp. 397–406 in Huxley, C.R. & Cutler, F.C. (Eds) Ant-Plant Interactions (Oxford Science Publications). Oxford University Press, Oxford.

299. Curiosités Entomologiques. 170 pp., Raymond Chabaud, Paris.

300. Distribution et plantes-hôtes de *Chrysolina banksi* (Fabricius, 1775) (Coleoptera, Chrysomelinae). Nouvelle Revue d’Entomologie (N.S.) 8(2): 151–157.

301. Chrysomelidae feeding on fungi, algae, lichens or bacteria. Chrysomela Newsletter 24: 6.

302. Le Doryphore (*Leptinotarsa decemlineata* (Say, 1824)) menace l’Asie. L’Entomologiste 47(1): 29–48.

303. Plantes et Fourmis: le Mutualisme brisé. La Recherche 22(233):792–793.

304. Les Fourmis et les Plantes. Un exemple de coévolution. Office pour les Insectes et leur Environnement, Revue Insectes 83(4):3–6.

**1992**

305. Le Bon Jardinier. La Maison Rustique, Paris. Articles sur Plantes carnivores et Bromeliaceae. Tomes 2 & 3. Articles dispersés entre p. 1221 et 2700.

306. Contribution à la taxonomie, la distribution et la biologie des *Chrysolina* nord-américains (Col. Chrys.). L’Entomologiste 48(1): 29–51.

307. Jolivet P, Petitpierre, E.: Notes on *Timarcha*Chrysomela Newsletter 26: 2.

308. Notes on *Coelomera*. Chrysomela Newsletter 26: 8.

309. Changements de couleur physiologiques chez les Cassides. Insectes 86(3): 2–4.

310. Le XIX° Congrès International d’Entomologie à Pékin. Insectes 87(4 ): 9.

**1993**

311. Les Plantes et les Fourmis. La Garance Voyageuse 21: 2–5.

312. Jolivet P, Vasconcellos-Neto, J.: Convergence chez les Plantes Carnivores. La Recherche 24(253): 456–458.

313. La Serra do Cipo au Brésil. Bulletin de Liaison de l’Association des Coleopteristes de La Region Parisienne17: 7–12.

314. Jolivet P, Salinas, P.J.: Ponte de *Coelomera cajennensis* (Fabricius,1787) dans la tige des *Cecropia*. Bulletin de la Société Entomologique de France 98(5): 472).

315. Mimétisme comportemental sous les Tropiques. Bulletin de Liaison de l’Association des Coleopteristes de La Region Parisienne18: 29–36.

**1994**

316. Jolivet, P.; Cox, M.L. & Petitpierre, E. (Eds) Novel Aspects of the Biology of Chrysomelidae. 582 pp., Kluwer Academic Publishers, Dordrecht.

317. Physiological Colour Changes in Tortoise Beetles. Pp 31- 335 in Novel Aspects of the Biology of Chrysomelidae (Nr. 316).

318. Mafra-Neto A, Jolivet, P.: Entomophagy in Chrysomelidae: adult *Aristobrotica angulicollis* (Erichson) feeding on adult meloids (Coleoptera). Pp. 171–178 in Novel Aspects of the Biology of Chrysomelidae (Nr. 316).

319. Vasconcellos-Neto J, Jolivet, P.: Cycloalexy among chrysomelid larvae. Pp. 303–309 in Novel Aspects of the Biology of Chrysomelidae (Nr. 316).

320. Figueira, J.; Vasconcellos-Neto J, Jolivet, P.: Une nouvelle plante protocarnivore: *Paepalanthus bromeloides* Silv. (Eriocaulaceae) au Brésil. Revue d’Écologie 49(1): 3–9.

321 Aptérisme et brachyptérisme chez les Coléoptères Chrysomélides ou le malheur de ne pas voler. Insectes 92(1): 25–27.

322. A propos d’*Anophthalmus hitleri* Scheibel,1937. Bulletin de Liaison de l’Association des Coleopteristes de La Region Parisienne 19: 17–20.

323. Dernières nouvelles de la progression du Doryphore: *Leptinotarsa decemlineata* (Say, 1824) (Col. Chrys.). L’Entomologiste 50(2): 105–111.

324. Jolivet P, Vasconcellos-Neto, J.: Apterous beetles in danger of extinction: the genus *Elytrosphaera* Stal. Chrysomela Newsletter 28: 4.

325. Mafra-Neto A, Jolivet, P.: The first record of Entomophagy in Chrysomelidae: Adult *Diabrotica angulicollis* (Erichson) feeding on the Meloid *Epicauta aterrima* (Klug) (Coleoptera). Chrysomela Newsletter 28: 5.

326. Remarks on the biology and biogeography of *Timarcha* (Chrysomelidae: Chrysomelinae). Pp. 85–97 in Furth, D.G. (ed.) Proceedings of the Third International Symposium on the Chrysomelidae, Beijing, 1992. Backhuys Publishers, Leiden:

**1995**

327. Réflexions sur les plantes-hôtes des Chrysomélides (Col.). L’Entomologiste 51(2): 77–93.

328. La Floride ou le Sunshine State. Un aperçu entomologique. Bulletin de Liaison de l’Association des Coleopteristes de La Region Parisienne 22: 31–35.

329. Jolivet P, Hawkeswood, T.J.: Host-plants of Chrysomelidae of the world, an essay about the relationships between the leaf-beetles and their food-plants. 281 pp., Backhuys Publishers, Leiden.

330. A status report on the species of *Timarcha* (Col. Chrys.). Insecta Mundi 9(1–2): 153–154.

331. Nomenclature et Nomenklatura. Bulletin de Liaison de l’Association des Coleopteristes de La Region Parisienne 24: 49–52.

332 Preliminary observations on the genus *Iscadida* Chevrolat, 1843, in South Africa (Coleoptera, Chrysomelidae). Nouvelle Revue d’Entomologie (N.S.) 12(4): 305–308.

333. A visit to the birth place of Jean-Henri Fabre. Chrysomela Newsletter 31: 11.

334. Jolivet P, Maes, J.-M.: A Biological Accident or a Strange case of Phoresy? (Coleoptera; Galerucinae: Lepidoptera; Danainae). Chrysomela Newsletter 30: 4

**1996**

335. Grobbelaar B, Jolivet, P.: Preliminary biological observations on the Southern African Chrysomeline genus *Iscadida* Chevrolat, 1843. Chrysomela Newsletter 31: 8–9.

336. Ants and Plants. An example of coevolution. 303 pp., Backhuys Publishers, Leiden.

337. Jolivet P, Maes, J.-M.: Un cas de cycloalexie chez un Curculionide: *Phelypera distigma* (Boheman) (Hyperinae) au Nicaragua. L’Entomologiste 52(3): 97–100.

338. Jolivet P, Cox, M.L. (Eds) Chrysomelidae Biology. vol. 1: The Classification, Phylogeny and Genetics. 444 pp., SPB Academic Publishers, Amsterdam.

339. id., vol. 2: Ecological Studies, 465 pp.

340. id. vol. 3: General Studies, 365 pp.

341. Mafra-Neto A, Jolivet, P.: Cannibalism in leaf-beetles. Pp 195–211 in no. 339, Chrysomelidae Biology..

342. Survol Entomologique du Nicaragua et du Panama. Bulletin de Liaison de l’Association des Coleopteristes de La Region Parisienne 27: 33–40.

343. Windsor, D.M. & Jolivet, P.: Aspects of the morphology and ecology of two Panamanian ant-plants, *Hoffmannia vesciculifera* (Rubiaceae) and *Besleria formicaria* (Gesneriaceae). Journal of Tropical Ecology 12: 835–842.

344. Le Fantôme de Lamarck et l’effet Baldwin. Bulletin de Liaison de l’Association des Coleopteristes de La Region Parisienne 28: 31–36.

**1997**

345. A la poursuite du *Timarcha* perdu: *Timarcha melitensis* Weise (Col. Chrys.). L’Entomologiste 52 (6): 203–211.

346. Plagiats et plagiaires en entomologie. Bulletin de Liaison de l’Association des Coleopteristes de La Region Parisienne 29: 49–52.

347. Biologie des Coléoptères Chrysomélides. 279 pp., Boubée Publishers, Paris.

348 Windsor D, Jolivet, P. Cycad-feeding by an ancient beetle, *Aulacoscelis* in Panama. Chrysomela Newsletter 34: 5.

349. *Timarcha djerbensis* Pic lives! Chrysomela Newsletter 34: 8.

350. Hawkeswood, T.J.; Takizawa H, Jolivet, P.: Observations on the biology and host-plants of the Australian tortoise beetle, *Cassida compuncta* (Boheman) with a description of the larva, pupa and adult (Insecta, Coleoptera, Chrysomelidae). Mauritiana (Altenburg) 16(2): 333–339.

351 Aiello A, Jolivet, P.: Myrmecophily in Keroplatidae (Diptera: Sciaroidea). Journal of the New York Entomological Society 104 (3–4): 226–230.

**1998**

352 Les nouveaux envahisseurs ou les Chrysomélides voyageurs. L’Entomologiste 54(1): 33–44.

353 Encore des «funny names» en Zoologie. Le Coléoptériste 32: 25–28.

354. Jurassic Park ou les Coléoptères des Cycadales. Le Coléoptériste 33: 77–85.

355. O tempora, o mores! Le Coléoptériste 33: 123.

356. Far Eastern Entomology. Chrysomela Newsletter 36: 8–9.

357. Manipulation du comportement chez les Fourmis et les Coléoptères sous l’influence de leurs parasites. L’Entomologiste 54(5): 211–222.

358. Host Plants of the Entomoscelina (Col. ChrysomelidaeChrysomelinae). Pp. 125–136 in Biondi, M.; Daccordi M, Furth, D.G. (Eds) Proceedings of the Fourth International Symposium on the Chrysomelidae. Museo Regional di Scienze Naturali, Atti, Torino.

359. de Macedo, M.V.; Vasconcellos-Neto J, Jolivet, P.: New biological data on the apterous beetle *Elytrosphaera lahtivirtai* Bechyné (Chrysomelidae: Chrysomelinae) and remarks on the biology and distribution of the genus. Pp. 271–279 in Biondi, M.; Daccordi M, Furth, D.G. (Eds) Proceedings of the Fourth International Symposium on the Chrysomelidae. Museo Regional di Scienze Naturali, Atti, Torino.

360. Vasconcellos-Neto J, Jolivet, P.: Are Brazilian species of *Elytrosphaera* (Col. Chrysomelidae), an apterous genus, threatened of extinction? Pp. 299–309 in Biondi, M.; Daccordi M, Furth, D.G. (Eds) Proceedings of the Fourth International Symposium on the Chrysomelidae. Museo Regional di Scienze Naturali, Atti, Torino.

**1999**

361. La menace des Insectes. Un nouveau casse-tête pour les Entomologistes: Les Bactéries des Insectes attaquent-elles aussi l’Homme? L’Entomologiste 55(2): 73–78.

362. Les Amours Compliquées des Coléoptères Chrysomélides. Le Coléoptériste 35: 29–36.

363. «Women’s Lib. chez les Insectes», ou quand le sexe faible devient le sexe fort (Col. Scirtidae, Lep. Nymphalidae & Lycaenidae). L’Entomologiste 55(3): 103–122.

364. Timarchophilia or Timarchomania. Reflexions on the genus *Timarcha* (Col. Chrys.). Nouvelle Revue d’Entomologie (N.S.) 16 (1): 11–18.

365. Sexual Behaviour Among Chrysomelidae. Pp. 391–409 in Cox, M.L. (ed.) Advances in Chrysomelidae Biology I. Backhuys Publishers., Leiden.

366. Du nouveau sur les *Metallotimarcha* Motschulsky (Col. Chrys.). Le Coléoptériste 37: 197–201.

367. Les espèces du genre *Aulacophora* Chevrolat sont-elles polyphages comme celles des *Diabrotica* Chevrolat? L’Entomologiste 55(6): 251–258.

368. Windsor, D.; Ness, J.; Gomez, L.D. & Jolivet, P.: Species of *Aulacoscelis* Duponchel and Chevrolat (Chrysomelidae) and *Nomotus* Gorham (Languriidae) feed on fronds of Central American cycads. Coleopterists Bulletin 53(3): 217–231.

369. In Memoriam: Ross Harold Arnett, Jr. (1919–1999). Nouvelle Revue d’Entomologie (N.S.) 16(2):195–196.

370. In Memoriam: Roy A. Crowson (1914–1999). Nouvelle Revue d’Entomologie (N.S.) 16(3): 291–294.

371. Disparition de deux grands coléoptéristes. Le Coléoptériste 37: 155–157.

372. Les Insectes ont-il des droits? Ou le Standing moral des Insectes. Le Coléoptériste 39: 103–111.

373. Courtship and Mating Behaviour among Leaf-Beetles. Pp. 115–125 in Sobti, R.C. & Yadav, J.S. (Eds) Some Aspects on the Insight of Insect Biology. Narendra Publishing House, New Delhi.

**2000**

374. Clark, S.M. & Jolivet, P.: *Timarcha* lives (in Montana)! Chrysomela Newsletter 38/39: 3.

375. Subaquatic Chrysomelidae. Abstracts of the XXI International Congress of Entomology, Iguaçu, Brazil 2: 1044.

376. In Memoriam: Jean Théodoridès (1926–1999). L’Entomologiste 56 (2): 77–80.

377. Verma, K.K. & Jolivet, P.: Phylogeny of Synetinae - reconsidered. Nouvelle Revue d’Entomologie (N. S.) 17(1): 35–49.

378. Second voyage entomologique au Nicaragua (28 Janvier-17 Février 2000). Le Coléoptériste 40: 175–180.

379. Crisomélids, una font d’inspiracio – Leaf Beetles, a source of inspiration. Editorial. Bolleti de la Societat d’Història Natural de les Balears 43: 9–13.

**2001**

380. What is a Chrysomelid? Nouvelle Revue Entomologie (N. S.): 18(2):135–146.

381.. Hawkeswood, T.J. & Jolivet, P.: Some notes on the biology and host plants of some Chrysomelidae (Coleoptera) from Australia. Entomologische Zeitschrift 111(9): 279–282.

382. Kalaichelvan, T.; Jolivet, P.; Verma, K.K. & Doguet, S. *Chaetocnema confinis* Crotch. (Col. Alticinae) in India. Nouvelle Revue Entomologie (N.S.) 18(3): 241–243.

383. Chaetocnema (Tlanoma) confinis Crotch, 1873. *Crop Protection Compendium*, Global Module 2000 edition. CD-ROM. CAB International, Wallingford, Oxon, U.K.

384. Les Plantes Myrmécophiles. Les Faits et les Théories. L’Entomologiste 57(1): 25–40.

385. Vers la Mondialisation des Chrysomélides? (Coleoptera). L’Entomologiste 57(3–4): 123–141.

386. Desportes-Livage I, Jolivet, P.: In Memoriam Jean Théodoridès (1926–1999). Parasite 8: 168.

387. High Altitude Chrysomelidae. Pp. 293–300 in Prakash, I. (ed) Ecology of Desert Environments. A Festschrift for Prof. J. L. Cloudsley-Thompson on his 80th Birthday. Scientific Publishers, Jodhpur.

388. A myrmecomorphic beetle which can be myrmecophilous: *Ctenostoma spinosum* Naviaux, 1998 (Col. Cicindelidae). Nouvelle Revue Entomologie (N.S.) 18(4): 315–316.

**2002**

389. Jolivet P, Verma, K.K.: Biology of Leaf-Beetles. 350 pp., Intercept, Andover.

390. Verma, K.K. & Jolivet, P.: Comments on “Spilopyrinae” (Col., Chrysomelidae). Nouvelle Revue d’Entomologie (N. S.) 19(2): 99–110.

391. Les Insectes souffrent-ils? Le Coléoptériste 5(2): 93–94.

392. Hawkeswood, T.J. & Jolivet, P.: Notes on the biology, distribution and host plants of *Oides circumdata* (Baly, 1861) and *Oides fryi* Clark, 1864 (ColeopteraChrysomelidae )from the rainforests of south-eastern Queensland, Australia. Entomologische Zeitschrift, Stuttgart 112(2): 51–52.

393. Poinar, jr. G.; Jolivet P, Grafteaux, A.: New Food-Plants provide clues for the origin and distribution of *Timarcha*. (Col. Chrysomelidae, Chrysomelinae). Lambillionea, Bruxelles 102(1): 103–109.

**2003**

394. Jolivet, P.; Verma, K.K. & Mille, C.: Biology and taxonomy of *Bohumiljania caledonica* (Jolivet) (Col., Chrysomelidae). Nouvelle Revue Entomologie (N.S.) 20(1): 3–22.

395. Subaquatic Chrysomelidae. Pp. 303–332 in Furth, D.G. (ed.) Special Topics in Leaf Beetle Biology – Proceedings of the Fifth International Symposium on the Chrysomelidae. Pensoft Publishers. Sofia - Moscow.

396. Hawkeswood, T.J. & Jolivet, P.: Notes on the biology and host plants of the Australian leaf beetle *Podagrica submetallica* (Blackburn) (Coleoptera: Chrysomelidae: Alticinae). Pp. 209–212 in Furth, D.G. (ed.) Special Topics in Leaf Beetle Biology – Proceedings of the Fifth International Symposium on the Chrysomelidae. Pensoft Publishers. Sofia - Moscow.

**2004**

397. Chapters: Cycloalexy; Inverted Copulation in Some Insects; Timarcha Latreille (ColeopteraChrysomelidaeChrysomelinae) in Capinera, J. (ed.) Encyclopedia of Entomology. 3 vols. Kluwer Academic Publishers, Dordrecht, 2004; pp. 654–656; 1208–1312; 2265–2269;

398. New Developments in the Biology of Chrysomelidae. Jolivet, P.; Santiago-Blay, J A. & Schmitt, M. (Eds). 804 pp., SPB Academic Publishing, The Hague.

399. Adaptations of Chrysomelidae (Coleoptera) from xeric regions. pp. 249–256 in no. 398 (ante).

400. Poinar, jr. G. & Jolivet, P.: Origin of *Timarcha*. Trophic relationships between the Old and the New World. pp. 281–290 in no. 398 (ante).

401. Verma, K.K. & Jolivet, P.: The primitive Eumolpinae and the Gondwana hypothesis. pp. 395–406 in no. 398 (ante).

402. Brachelytry among Chrysomelidae (Coleoptera). Lambilionea, Brussels 105(3): 371–384.

403. Evolution des Spilopyrinoides du Chili à la Nouvelle Calédonie (Col. Chrysomelidae). (Conférence du 17 Février 2004). Le Coléoptériste 7(2): 79–86.

404. Un mois de Mai mémorable en Amérique du Nord. Insectes 135: 3–7.

405. Fabreries in Fabreland. Chrysomela Newsletter 43: 17–18.

406. In Memoriam. Michio Chûjô. 1908–2004. Stephan Iablokoff-Khnzorian. 1904–1995. Chrysomela Newsletter 44:10, 2004.

407. Mille, Chr. & Jolivet, P.: Insect collecting in Mont Do, in New Caledonia. Chrysomela Newsletter 44: 23–24 & Le Coléoptériste 8(2): 111–112.

408. Xavier Montrouzier (1820–1897), a Pioneer of Neo Caledonian entomology. Chrysomela Newsletter 44: 25–26.

**2005**

409. Verma, K. K.; Gomez-Zurita, J.; Jolivet P, Vig, K.: Biology of *Eupales ulema* (Germar,1813) and its taxonomic placement among Eumolpinae (ColeopteraChrysomelidae). Nouvelle Revue d’Entomologie (N.S.): 22(2): 155–164.

410. Gomez-Zurita, J.; Jolivet P, Vogler, A.: Molecular Systematics of Eumolpinae and the relationships with Spilopyrinae (Coleoptera, Chrysomelidae). Molecular Phylogenetics and Evolution 34(3): 584–600.

411. Au berceau des Fabreries. Les Amis de Jean-Henri Fabre, Saint-Léons 19: 2–3.

412. Verma, K.K. & Jolivet, P.: In defence of retaining *Syneta* Dejean in its own subfamily, Synetinae (Coleoptera: Chrysomelidae). Nouvelle Revue d’Entomologie (N.S.) 22(3): 277–280.

413. Jolivet P, Verma, K.K.: Fascinating Insects. Some aspects of insect life. 310 pp., Pensoft Publishers, Sofia - Moscow.

414. Les vers luisants de la Nouvelle-Zélande ou quand un rêve se réalise. Insectes 138: 29–33.

415. Jolivet, P.; Verma, K.K. & Mille, C.: New observations on the biology of Chrysomelidae (Coleoptera) of New Caledonia and description of two new chrysomelid species from the main island. Revue Française d’Entomologie (N.S.) 27(2): 63–72.

416. Xavier Montrouzier (1820–1897), the pioneer entomologist of New Caledonia, and the New Caledonian Chrysomelidae. Nouvelle Revue d’Entomologie (N.S.) 22(2): 183–186.

417. Jolivet P, Jolivet, M.: A New Caledonian Cycad Pictorial. The Cycad Newsletter 28(2): 3.

418. Jolivet P, Mille, Chr.: Rapport de Mission entomologique sur le Mont Humboldt (du 17 au 18 février 2005). Institut Agronomique néo-Calédonien. Station de Recherches de Pocquereux. 20.09.2005: 6 pp.

419. Cycads and Beetles: Recent Views on Pollination. The Cycad Newsletter 28(3–4): 3–7.

**2006**

420. Verma, K.K. & Jolivet, P.: On phyletic closeness between South American and New Caledonian spilopyrini (Chrysomelidae, Eumolpinae, Tribe Spilopyrini). Bonner zoologische Beiträge 54(4): 297–303.

421. Mémoires Entomologiques. Paramémoires d’un Timarchophile. 350 pp., Pensoft. Sofia-Moscow.

422. Nomenclature and Nomenklatura. Taxonomy, Taxinomy or Systematics. Nouvelle Revue d’Entomologie (N.S.) 22(4): 355–360.

423. François Cohic, entomologiste (1921–1992). Nouvelle Revue d’Entomologie (N.S.) 23(1): 75–78.

424. Julien Achard (1881–1925). Le Coléoptériste 8(3): 206–207.

**2007**

425. Sur les traces de Wollaston…. et bredouille. Le Coléoptériste 10(2): 131–132.

426. Jolivet, P.; Verma, K.K. & Mille, C.: New species of Eumolpinae from the genus *Dematochroma* Baly, 1864 and *Taophila* Heller, 1916 from New Caledonia. Revue Française d’Entomologie (N.S.) 29(1): 33–47.

427. Jean Risbec (1895–1964). Un pionnier de l’entomologie appliquée en Nouvelle-Calédonie. Nouvelle Revue d’Entomologie (N.S.) 24(1): 84–86.

428. Jolivet, P.; Verma, K.K. & Mille, C: New species of *Dematochroma* and *Colaspoides* from New Caledonia. (Coleoptera, Chrysomelidae, Eumolpinae). Nouvelle Revue d’Entomologie (N.S.) 24(3): 195–200.

429. Jolivet, P.; Verma, K.K. & Mille, C: New species of *Dematochroma* from Lord Howe and Norfolk islands (Coleoptera, Chrysomelidae, Eumolpinae). Nouvelle Revue d’Entomologie (N.S.) 23(4): 327–332.

430. Julien Achard (1881–1925). Chrysomela Newsletter 47: 6.

431. Chapters: Cycloalexy; Timarcha; Inverted Copulation; Sweetpotato Flea Beetle, Chaetocnema confinis; Hypertely; Brachelytry; in Capinera, J. (ed.) Encyclopedia of Entomology. 2nd ed. Springer Science Publishers, Dordrecht. 2008. [pp. 1139–1140; 3820–3823; 2041–2044; 3640–3642; 1897–1901; 555–560.]

432. Jolivet, P., Verma, K. K. & Mille, Chr. New genera and species of Eumolpinae (Coleoptera: Chrysomelidae) from New Caledonia. Revue Française d’Entomologie (N.S.) 29(2–3): 77–92.

433. Poinar jr., G.; Zoia S, Jolivet, P.: Mermithid parasites of *Timarcha*. Chrysomela Newsletter 48: 6.

434. Hypertélie: mimétisme, signaux sexuels ou moyens de défense. Un dilemme chez les Insectes: vrai ou faux concept? L’Entomologiste 63(2): 57–66.

435. Le principe de Baldwin ou l’effet Baldwin en biologie. Une bonne explication ou une échappatoire des darwinistes? L’Entomologiste 63(6): 309–318.

436. Jean Lhoste. Chrysomela Newsletter 49: 16.

**2008**

437. Léon Croizat (1894–1982). Hommes et Plantes 64: 10–13.

438. Jolivet P, Poinar jr., G.: Parasites, commensals and phoretics of *Timarcha* (Coleoptera: Chrysomelidae). Papers Celebrating the 80^th^ Birthday of Professor Andrzej Warchalowski. Genus 18(4): 589–596.

439. Nocturnal Hunting Grounds. Chrysomela Newsletter 50–51: 4–5.

440. Doguet S, Jolivet, P.: In memoriam Michel Bergeal, Nicole Berti, Enrique Balcells y Rocamora. Chrysomela Newsletter 50–51: 18.

441. La faune entomologique en Nouvelle-Calédonie. Le Coléoptériste 11(1): 35–47.

442. Jolivet P, Verma, K.K. Eumolpinae- a widely distributed and much diversified subfamily of leaf beetles (Coleoptera, Chrysomelidae). Terrestrial Arthropod Reviews 1: 3–37.

443. Baldwin effect and Phenotypic plasticity. Abstracts of the XXth International Congress of Zoology, Paris-26–29 August 2008: 107.

444. SOS Orsodacninae. Le Coléoptériste 11(2): 102.

445. Jolivet, P.; Santiago-Blay J, Schmitt, M. (Eds) Research on Chrysomelidae, Volume 1. 430 pp., Brill Publishers. Leiden - Boston.

446. Beenen R, Jolivet, P.: Classification and habitat of brachelytrous Chrysomelidae (Coleoptera). pp. 161–173 in no. 445 (cf. ante).

447. Jolivet P, Verma, K.K.: On the origin of the Chrysomelid Fauna of New Caledonia. pp. 309–319 no. 445 (cf. ante).

**2009**

448. Jolivet, P.; Santiago-Blay J, Schmitt, M. (Eds) Research on Chrysomelidae, Volume 2. 299 pp., Brill Publishers, Leiden - Boston.

449. Jolivet P, Verma, K.K.: Biogeography and Biology of the New Caledonian Chrysomelidae (Coleoptera). pp. 211–223 in no. 448 (Proceedings of the 7^th^ International Symposium on the Chrysomelidae).

450. Léon Croizat (1894–1982), chevalier des temps modernes et pionnier de la Biogéographie. Le Coléoptériste 12(1): 36–40.

451. Comment on the proposed conservation of Aulacoscelinae Chapuis 1874 (Insecta: Coleoptera: Orsodacnidae or Chrysomelidae) (Case 3398). Bulletin of Zoological Nomenclature 66(1): 72.

452. Eloge de Jean-Baptiste de Lamarck (1744–1829). Le Coléoptériste 12(3): 168–171.

**2010**

453. Jolivet P, Verma, K.K.: Good morning Gondwana. Annales de la Société Entomologique de France. Annales de la Société Entomologique de France (N.S.) 46(1–2): 53–61.

454. Jolivet, P.,Verma, K.K. & Mille, C.: Eumolpinae (Coleoptera) recently collected in New Caledonia and Vanuatu. Nouvelle Revue d’Entomologie (N.S.) 26(1): 3–17.

455. Evolution.Vers une nouvelle synthèse. La Plasticité Phénotypique. Le Coléoptériste 12(2): 106–119

456. Réflexions impromptues sur l’Evolution. L’Entomologiste 65(5): 239–248.

457. Les défis de l’Évolution. L’Entomologiste 66(2): 93–103.

458. Jolivet, P.; Verma, K.K. & Mille, C.: New Eumolpinae (Coleoptera: Chrysomelidae) from Mt. Panié, New Caledonia. Nouvelle Revue d’Entomologie (N.S.) 26(4): 333–340.

459. In memoriam: Rémy Chauvin (1913–2009). L’Entomologiste 66(1): 5–6.

460. Vert, jaune, blanc ou rouge... Pourquoi les feuilles changent-elles de couleur? Incidence sur les Insectes. L’Entomologiste 66(3): 153–164.

461. Jean Lhoste: French coleopterist. Chrysomela Newsletter 52: 6.

462. Ad Augusta per Angusta. Les Adaptations spécialisées de la Vie dans l’Obscurité. Le Coléoptériste 13(2): 129–141 & 13(3):162–171.

463. Eloge de Charles Darwin (1809–1882). Le Coléoptériste 13(1): 11–16.

464. Gomez-Zurita, J.; Cardoso, A.; Jurado-Rivera, J.A.; Jolivet, P.; Cazères S, Mille, C.: Discovery of new species of New Caledonian *Arsipoda* Erichson 1842 (Coleoptera, Chrysomelidae) and insights on their ecology and evolution using DNA markers. Journal of Natural History 44(41–42): 2557–2579.

465. En marge de l’évolution! De nouvelles idées, de nouvelles hypothèses, de nouvelles propositions! L’Entomologiste 66(4): 199–213.

466. Évolution et coévolution. L’Entomologiste 66(5–6): 263–280.

**2011**

467. Les Arcanes de l’Évolution: quelques questions nouvelles concernant l’Évolution. L’Entomologiste 67(4): 199–215.

468. Jolivet P, Verma, K.K.: Southern biota and the Gondwana hypothesis. Nouvelle Revue d’Entomologie (N.S.) 27(1): 53–69.

469. Chronique ordinaire de l’Évolution. L’Entomologiste 68(2): 79–102.

470. Jolivet, P.; Verma, K.K. & Mille, C.: Eumolpinae (Coleoptera, Chrysomelidae) recently collected in New Caledonia: A consideration of local geographic intraspecific variability in *Bohumiljania humboldti*. Revue Française d’Entomologie (N.S.) 32(3–4): 141–149.

471. Jolivet P, Verma, K.K.: Reflexions on cycloalexy among Chrysomelidae (Coleoptera). Nouvelle Revue d’Entomologie (N.S.) 27(4): 311–329.

472. Jolivet, P.; Santiago-Blay J, Schmitt, M. (Eds). Research on Chrysomelidae, Volume 3 (Zookeys 157). 179 pp., Pensoft, Sofia-Moscow.

473. Jean Risbec (1895–1964), un entomologiste oublié. Le Coléoptériste 14(3): 211–215.

**2012**

474. Jolivet P, Verma, K.K.: Vicariance and short-distance dispersals: Main drivers of the southern biogeography. Nouvelle Revue d’Entomologie (N.S.) 28(1): 37–45.

475. Panorama de l’Évolution. L’Entomologiste 68(4): 217–238.

476. In memoriam. Neal Smith (1937–2012). Humaniste, ornithologiste, botaniste et entomologiste. L’Entomologiste 68(5): 265–266.

477. Santiago-Blay, J.A.; Jolivet J, Verma, K.K.: A natural history of conspecific aggregations in terrestrial arthropods, with emphasis on cycloalexy in leaf beetles (Coleoptera: Chrysomelidae). Terrestrial Arthropod Reviews 5(3–4): 289–355.

**2013**

478. Verma, K.K.; Jolivet P, Saxena, R.: Species and Speciation. Biological Forum - An International Journal 5(1): 42–49.

479. Verma, K.K.; Jolivet P, Saxena, R.: “Retournement” of the aedeagus in Curculionidae (Coleoptera, Curculionoidea). Insecta Mundi 0300: 1–5.

480. Nouvelles données sur l’Évolution. L’Entomologiste 69(3): 157–178.

481. Verma, K.K.; Jolivet P, Saxena, R.: More on Speciation-A View. Biological Forum -An International Journal 5(2): 8–10.

482. Jolivet, P.; Santiago-Blay J, Schmitt, M. (Eds). Research on Chrysomelidae, Volume 4 (ZooKeys 332). 231 pp., Pensoft, Sofia-Moscow.

483. Jolivet, P.; Verma, K.K. & Mille, Chr.: New and known leafbeetles collected in Mont Panié in 2010 (Coleoptera, Chrysomelidae, Eumolpinae). Nouvelle Revue d’Entomologie (N.S.) 29 (3–4): 141–154.

484. Jolivet, P.; Verma, K.K. & Saxena, R.: Speciation- a continuum. Nouvelle Revue d’Entomologie (N.S.) 29(3–4): 162–166.

**2014**

485. Jolivet, P.; Poinar, jr. G, Verma, K.K.: *Timarcha* Latreille: A strange beetle and a living fossil. Terrestrial Arthropod Reviews 7: 3–20.

486. Évolution, non pas Révolution. L’Entomologiste 70(1): 15–38.

487. Jolivet, P., Lawrence, J, F, Verma, K.K. & Slipinski, A.: Eumolpinae. Pp. 217–225 in Leschen, R.A.B. & Beutel, R.G. (Eds). Handbook of Zoology, Coleoptera 3. Walter de Gruyter, Berlin - Boston.

488. Jolivet, P., Lawrence, J,F, Reid C.M. & Verma, K.K.: Spilopyrinae. Pp. 271–277 in Leschen, R.A.B. & Beutel, R.G. (Eds) Handbook of Zoology, Coleoptera 3. Walter de Gruyter, Berlin - Boston.

489. Les étranges migrations des Uraniides (LepidopteraGeometroideaUraniidae). L’Entomologiste 70(3): 129–139.

490. Après Linné, Buffon et Darwin. L’évolution selon Princeton. Le Coléoptériste 17(1): 26–27.

**2015**

491. Jolivet, P.: Nouvelle fenêtre sur l’évolution. L’Entomologiste 71(4): 221–242.

492. Jolivet, P. 2015. A Flashback: The Malaria Advisory Teams. WHO Quarterly News. Geneva. July 2015, 100: 13–14.

493. Jolivet, P.: Nouvelles réflexiones sur l’évolution. Entomologie et biologie. L’Entomologiste 71(5): 333–354.

**In Press**

494. Mille, Chr. & Jolivet, P.: Catalogue Provisoire des Chrysomelidae de la Nouvelle Calédonie (Coleoptera).

